# DNA barcoding evidence for the North American presence of alfalfa cyst nematode, *Heterodera medicaginis*

**DOI:** 10.21307/jofnem-2019-016

**Published:** 2019-04-24

**Authors:** Thomas Powers, Andrea Skantar, Tim Harris, Rebecca Higgins, Peter Mullin, Saad Hafez, Zafar Handoo, Tim Todd, Kirsten Powers

**Affiliations:** 1 University of Nebraska-Lincoln, Lincoln, NE, 68583-0722; 2 Mycology and Nematology Genetic Diversity and Biology Laboratory (MNGDBL), USDA, Beltsville, MD; 3 Department of Plant Pathology, University of Idaho, Boise, ID; 4 Kansas State University, Manhattan, Kansas

**Keywords:** Alfalfa cyst nematode, COI DNA barcode, Detection, Diagnosis, *Heterodera medicaginis*, Taxonomy

## Abstract

Specimens of *Heterodera* have been collected from alfalfa fields in Kearny County, Kansas & Carbon County, Montana. DNA barcoding with the COI mitochondrial gene indicate that the species is not *Heterodera glycines*, soybean cyst nematode, *H. schachtii*, sugar beet cyst nematode, or *H. trifolii*, clover cyst nematode. Maximum likelihood phylogenetic trees show that the alfalfa specimens form a sister clade most closely related to *H. glycines*, with a 4.7% mean pairwise sequence divergence across the 862 nucleotides of the COI marker. Morphological analyses of juveniles and cysts conform to the measurements of *H. medicaginis*, the alfalfa cyst nematode originally described from the USSR in 1971. Initial host testing demonstrated that the nematode reproduced on alfalfa, but not on soybeans, tomato, or corn. Collectively, the evidence suggests that this finding represents the first record of *H. medicaginis* in North America. Definitive confirmation of this diagnosis would require COI sequence of eastern European isolates of this species.

Alfalfa cyst nematode, *Heterodera medicaginis* (Kirjanova & Krall, 1971) was originally described from the USSR in 1971 and redescribed by Gerber and Maas (1982). The redescription added information missing in the original description regarding juvenile and male stages (Gerber and Maas, 1982). Host testing was conducted for the redescription that included 20 plant species from 7 plant families. These tests included *Glycine max*, *Medicago lupulina*, *M. sativa*, *Phaseolus vulgaris*, *Pisum sativum*, *Trifolium pratense*, *T. repens*, *T. subterraneum*, *Vicia faba*, *V. sativa*, *Vigna sinensis*, *Galeopsis tetrahit*, *Dianthus plumarius*, *Spergula arvensis*, *Stellaria media*, *Beta vulgaris*, *Spinacia oleracea*, *Brassica oleracea*, *B. rapa*, *Rumex crispus*, and *Hebe andersonii*. These host trials concluded that *Heterodera medicaginis* was only able to complete reproduction on alfalfa, *Medicago sativa.* The redescription included a narrative that provided morphological traits that could help discriminate the species from other members of the *H. schachtii* group. To discriminate the J2 stages of *H*. *medicaginis* and *H. glycines* Ichinohe 1952, it was determined that *H. medicaginis* possessed a longer stylet (25 µm vs 23 µm in *H. glycines*). Similarly, adult males could be identified by a longer stylet (29 µm vs 27 µm in *H. glycines*). The cyst stage of *H. medicaginis* was also notable because of its ‘weakly developed, unbranched underbridge’ (Gerber and Maas, 1982). Following the redescription, a DNA sequence of the internal transcribed spacer region was submitted to GenBank (Subbotin et al., 2001).

According to Subbotin et al. (2010), *H*. *medicaginis* is known from the Russian regions of Rostov, Stavropol Territory, Krasnodar Territory, and Kabardino Balkaria, as well as from Ukraine, Kazakhstan, and Uzbekistan (Baidulova, 1981). Furthermore, unidentified cyst nematodes were observed on lucerne roots in Poland (Brzeski, 1998).

During the last five years, there have been unpublished reports of a cyst species reproducing on alfalfa (https://nematode.unl.edu/hetemedic.htm) in the Great Plains state of Kansas in the US. A follow-up of these reports, utilizing morphology, host trials, and DNA barcoding using the mitochondrial gene COI along with ITS1, the transcribed spacer region between 18S and 5.8S of the nuclear ribosomal repeat region, and the heat shock protein gene Hsp90 provide supporting evidence that *Heterodera medicaginis* is present in the US.

## Materials and methods

### Nematode collections

The original North American collections of suspect alfalfa cyst specimens were made in western Kansas from alfalfa fields that bordered the Arkansas River near the city of Lakin, Kearny County, KS (Table 1). Some soil was sent to the Kansas State University Diagnostic Laboratory, the University of Idaho Nematology Laboratory, and cysts were sent to the University of Nebraska and the USDA Mycology and Nematology Genetic Diversity and Biology Laboratory (MNGDBL) at Beltsville, Maryland. Additionally, several cysts containing eggs and juveniles were isolated from an alfalfa planting in Carbon County, Montana during a Cooperative Agricultural Pest Survey in 2006 (Table 1).

**Table 1:** Collection data for specimens used in this study. Specimens 1 to 15 were examined as fixed specimens.

Specimen ID	Species	Locality	Host	Marker	GenBank Accession No.
1	*H. medicaginis*	Kearny County, Kansas	Alfalfa		N/A
2	*H. medicaginis*	Kearny County, Kansas	Alfalfa		N/A
3	*H. medicaginis*	Kearny County, Kansas	Alfalfa		N/A
4	*H. medicaginis*	Kearny County, Kansas	Alfalfa		N/A
5	*H. medicaginis*	Kearny County, Kansas	Alfalfa		N/A
6	*H. medicaginis*	Kearny County, Kansas	Alfalfa		N/A
7	*H. medicaginis*	Kearny County, Kansas	Alfalfa		N/A
8	*H. medicaginis*	Kearny County, Kansas	Alfalfa		N/A
9	*H. medicaginis*	Kearny County, Kansas	Alfalfa		N/A
10	*H. medicaginis*	Kearny County, Kansas	Alfalfa		N/A
11	*H. medicaginis*	Kearny County, Kansas	Alfalfa		N/A
12	*H. medicaginis*	Kearny County, Kansas	Alfalfa		N/A
13	*H. medicaginis*	Kearny County, Kansas	Alfalfa		N/A
14	*H. medicaginis*	Kearny County, Kansas	Alfalfa		N/A
15	*H. medicaginis*	Kearny County, Kansas	Alfalfa		N/A
P169028	*H. medicaginis*	Kearny County, Kansas	Alfalfa	18S	AY912048
N838	*H. schachtii*	Goshen County, Wyoming	Soybean	COI	MK093062
N839	*H. schachtii*	Goshen County, Wyoming	Soybean	COI	MK093063
N864	*H. schachtii*	Goshen County, Wyoming	Soybean	COI	MK093064
N4143	*Meloidodera* sp.	Big Thicket National Preserve, Texas	Beech	COI	MK093163
N4178	*Meloidodera* sp.	Big Thicket National Preserve, Texas	Water oak	COI	MK093159
N7083	*H. avenae*	Rio Grande County, Colorado	Barley	COI	MK093164
N7095	*H. medicaginis*	Kearny County, Kansas	Alfalfa	COI	MK093160
N7096	*H. medicaginis*	Kearny County, Kansas	Alfalfa	COI	MK093162
N7243	*H. medicaginis*	Kearny County, Kansas	Alfalfa	COI ITS1	MK093168 MK093180
N7244	*H. medicaginis*	Kearny County, Kansas	Alfalfa	COI ITS1	MK093169 MK093181
N7245	*H. medicaginis*	Kearny County, Kansas	Alfalfa	COI	MK093170
N7246	*H. medicaginis*	Kearny County, Kansas	Alfalfa	COI ITS1	MK093171 MK093182
N7247	*H. medicaginis*	Kearny County, Kansas	Alfalfa	COI	MK093172
N7248	*H. trifolii*	Rio Arriba County, New Mexico	Vineyard	COI	MK093173
N7250	*H. trifolii*	Rio Arriba County, New Mexico	Vineyard	COI ITS1	MK093174 MK093183
N7251	*H. avenae*	Akershus, Norway	Oats	COI	MK093175
N7253	*H. avenae*	Akershus, Norway	Oats	COI	MK093176
N8306	*H. schachtii*	Platte County, Wyoming	Bean	COI	MK093165
N8876	*H. medicaginis*	Kearny County, Kansas	Alfalfa	COI	MK093166
N8877	*H. medicaginis*	Kearny County, Kansas	Alfalfa	COI	MK093161
N8881	*Heterodera* sp.	Dąbrówka, Poland	Peat meadow	COI	MK093167
P119063	*H. medicaginis*	Carbon County, Montana	Alfalfa	COI	MK093053
P119064	*H. schachtii*	Big Horn County, Montana	Alfalfa	COI	MK093054
P125039	*H. schachtii*	Platte County, Wyoming	Bean	COI	MK093055
P125063	*H. schachtii*	Laramie County, Wyoming	Sugar beet	COI	MK093056
P145076	*H. schachtii*	Platte County, Wyoming	Sugar beet	COI	MK093057
P150049	*H. avenae*	Montana	Barley	COI	MK093058
P150069	*Vittatidera zeaphila*	Tennessee	Corn	COI	MK093060
P164043	*H. avenae*	Montana	Barley	COI	MK093059
P200034	*Cactodera* sp.	Sheridan County, Nebraska	Potatoes-wheat	COI	MK093061
P231089	*H. glycines*	Unknown	Soybean	COI	MK093049
P213017	*H. glycines*	Unknown	Soybean	COI	MK093050
P231088	*H. glycines*	Unknown	Soybean	COI	MK093051
P231091	*H. schachtii*	Unknown	Soybean	COI	MK093052
P243062	*H. glycines*	Richardson County, Nebraska	Soybean	COI	MK093086
P243063	*H. glycines*	Richardson County, Nebraska	Soybean	COI	MK093087
P243064	*H. glycines*	Richardson County, Nebraska	Soybean	COI	MK093088
P243069	*H. glycines*	Seward County, Nebraska	Soybean	COI	MK093077
P243080	*H. glycines*	Lancaster County, Nebraska	Soybean	COI	MK093078
P243083	*H. glycines*	Lancaster County, Nebraska	Soybean	COI	MK093079
P243085	*H. glycines*	Cuming County, Nebraska	Soybean	COI	MK093080
P243086	*H. glycines*	Cuming County, Nebraska	Soybean	COI	MK093081
P243094	*H. glycines*	Nebraska	Soybean	COI	MK093084
P243096	*H. glycines*	Nebraska	Soybean	COI	MK093085
P244003	*H. glycines*	Cedar County, Nebraska	Soybean	COI	MK093095
P244004	*H. glycines*	Cedar County, Nebraska	Soybean	COI	MK093096
P244020	*H. glycines*	Holt County, Nebraska	Soybean	COI	MK093089
P244021	*H. glycines*	Holt County, Nebraska	Soybean	COI	MK093090
P244041	*H. glycines*	Buffalo County, Nebraska	Soybean	COI	MK093093
P244042	*H. glycines*	Buffalo County, Nebraska	Soybean	COI	MK093094
P244060	*H. glycines*	Burt County, Nebraska	Soybean	COI	MK093091
P244062	*H. glycines*	Burt County, Nebraska	Soybean	COI	MK093092
P244065	*H. glycines*	Stanton County, Nebraska	Soybean	COI	MK093082
P244066	*H. glycines*	Stanton County, Nebraska	Soybean	COI	MK093083
P244072	*H. glycines*	Cass County, Nebraska	Soybean	COI	MK093097
P244074	*H. glycines*	Cass County, Nebraska	Soybean	COI	MK093098
P244076	*H. schachtii*	Big Horn County, Montana	Pinto bean	COI	MK093065
P244077	*H. schachtii*	Big Horn County, Montana	Pinto bean	COI	MK093066
P244078	*H. schachtii*	Big Horn County, Montana	Black bean	COI	MK093067
P244079	*H. schachtii*	Big Horn County, Montana	Black bean	COI	MK093068
P244081	*H. schachtii*	Park County, Wyoming	Pinto bean	COI	MK093069
P244082	*H. schachtii*	Park County, Wyoming	Pinto bean	COI	MK093070
P244084	*H. schachtii*	Platte County, Wyoming	Pinto bean	COI	MK093071
P244085	*H. schachtii*	Platte County, Wyoming	Pinto bean	COI	MK093072
P244086	*H. schachtii*	Platte County, Wyoming	Pinto bean	COI	MK093073
P244087	*H. schachtii*	Platte County, Wyoming	Pinto bean	COI	MK093074
P244088	*H. schachtii*	Platte County, Wyoming	Pinto bean	COI	MK093075
P244089	*H. schachtii*	Platte County, Wyoming	Pinto bean	COI	MK093076
P245019	*H. glycines*	Alabama	Soybean	COI	MK093127
P245023	*H. glycines*	Alabama	Soybean	COI	MK093128
P245025	*H. glycines*	Alabama	Soybean	COI	MK093129
P245027	*H. glycines*	Alabama	Soybean	COI	MK093130
P245029	*H. glycines*	Alabama	Soybean	COI	MK093131
P245033	*H. glycines*	Alabama	Soybean	COI	MK093119
P245035	*H. glycines*	Alabama	Soybean	COI	MK093132
P245038	*H. glycines*	Alabama	Soybean	COI	MK093133
P245041	*H. glycines*	Alabama	Soybean	COI	MK093120
P245043	*H. glycines*	Alabama	Soybean	COI	MK093134
P245044	*H. glycines*	Cedar County, Nebraska	Soybean	COI	MK093109
P245046	*H. glycines*	Cedar County, Nebraska	Soybean	COI	MK093110
P245089	*H. glycines*	Oconee County, Georgia	Soybean	COI	MK093111
P245090	*H. glycines*	Oconee County, Georgia	Soybean	COI	MK093112
P245094	*H. glycines*	Burke County, Georgia	Soybean	COI	MK093135
P245095	*H. glycines*	Burke County, Georgia	Soybean	COI	MK093136
P246006	*H. glycines*	Boone County, Missouri	Soybean	COI	MK093121
P246008	*H. glycines*	Boone County, Missouri	Soybean	COI	MK093137
P246010	*H. glycines*	Livingston County, Missouri	Soybean	COI	MK093138
P246014	*H. glycines*	Knox County, Missouri	Soybean	COI	MK093113
P246016	*H. glycines*	Knox County, Missouri	Soybean	COI	MK093114
P246018	*H. glycines*	Atchison County, Missouri	Soybean	COI	MK093139
P246021	*H. glycines*	Atchison County, Missouri	Soybean	COI	MK093122
P246033	*H. glycines*	Fulton County, Ohio	Soybean	COI	MK093123
P246035	*H. glycines*	Fulton County, Ohio	Soybean	COI	MK093140
P246042	*H. glycines*	Sandusky County, Ohio	Soybean	COI	MK093124
P246044	*H. glycines*	Sandusky County, Ohio	Soybean	COI	MK093115
P246047	*H. glycines*	Sandusky County, Ohio	Soybean	COI	MK093116
P247094	*H. glycines*	Lee County, Arkansas	Soybean	COI	MK093141
P247096	*H. glycines*	Lee County, Arkansas	Soybean	COI	MK093142
P247099	*H. glycines*	Lee County, Arkansas	Soybean	COI	MK093143
P248001	*H. glycines*	Lee County, Arkansas	Soybean	COI	MK093144
P248004	*H. glycines*	Washington County, Arkansas	Soybean	COI	MK093145
P248006	*H. glycines*	Washington County, Arkansas	Soybean	COI	MK093146
P248009	*H. glycines*	McLeod County, Minnesota	Soybean	COI	MK093147
P248010	*H. glycines*	McLeod County, Minnesota	Soybean	COI	MK093125
P248013	*H. glycines*	Wilken County, Minnesota	Soybean	COI	MK093117
P248016	*H. glycines*	Wilken County, Minnesota	Soybean	COI	MK093118
P248017	*H. glycines*	Nicollet County, Minnesota	Soybean	COI	MK093148
P248019	*H. glycines*	Nicollet County, Minnesota	Soybean	COI	MK093149
P248023	*H. glycines*	Dodge County, Minnesota	Soybean	COI	MK093099
P248024	*H. glycines*	Dodge County, Minnesota	Soybean	COI	MK093100
P248026	*H. glycines*	Red Lake County, Minnesota	Soybean	COI	MK093101
P248027	*H. glycines*	Red Lake County, Minnesota	Soybean	COI	MK093102
P248029	*H. glycines*	Redwood County, Minnesota	Soybean	COI	MK093150
P248031	*H. glycines*	Redwood County, Minnesota	Soybean	COI	MK093151
P248035	*H. glycines*	Dakota County, Minnesota	Soybean	COI	MK093103
P248036	*H. glycines*	Dakota County, Minnesota	Soybean	COI	MK093104
P248040	*H. glycines*	Waseca County, Minnesota	Soybean	COI	MK093105
P248041	*H. glycines*	Waseca County, Minnesota	Soybean	COI	MK093106
P248058	*H.* cf. *urticae*	Faulkner County, Arkansas	Chickweed	COI	MK093155
P248059	*H.* cf. *urticae*	Faulkner County, Arkansas	Chickweed	COI	MK093156
P248060	*H. trifolii*	Washington County, Arkansas	Clover	COI	MK093157
P248061	*H. trifolii*	Washington County, Arkansas	Clover	COI	MK093158
P248066	*H. glycines*	Gentry County, Missouri	Soybean	COI	MK093107
P248068	*H. glycines*	Gentry County, Missouri	Soybean	COI	MK093108
P248071	*H. glycines*	Mercer County, Missouri	Soybean	COI	MK093152
P248073	*H. glycines*	Mercer County, Missouri	Soybean	COI	MK093153
P248086	*H. glycines*	Sussex County, Delaware	Soybean	COI	MK093154
P248087	*H. glycines*	Sussex County, Delaware	Soybean	COI	MK093126
105A13	*H. medicaginis*	Kearny County, Kansas	Alfalfa	COI	MK093177
105A14	*H. medicaginis*	Kearny County, Kansas	Alfalfa	COI	MK093178
105A15	*H. medicaginis*	Kearny County, Kansas	Alfalfa	COI	MK093179
Hsp90_3530	*H. medicaginis*	Kearny County, Kansas	Alfalfa	Hsp90	MH798843
Hsp90_3531	*H. medicaginis*	Kearny County, Kansas	Alfalfa	Hsp90	MH798844
Hsp90_3532	*H. medicaginis*	Kearny County, Kansas	Alfalfa	Hsp90	MH798845
Hsp90_3533	*H. medicaginis*	Kearny County, Kansas	Alfalfa	Hsp90	MH798846
Hsp90_2996	*H. trifolii*	Alexandria, Egypt	Sugar beet	Hsp90	MK095224
Hsp90_2997	*H. trifolii*	Alexandria, Egypt	Sugar beet	Hsp90	MK095225
Hsp90_2994	*H. trifolii*	Alexandria, Egypt	Sugar beet	Hsp90	MK095222
Hsp90_2995	*H. trifolii*	Alexandria, Egypt	Sugar beet	Hsp90	MK095223
Hsp90_2998	*H. trifolii*	Alexandria, Egypt	Sugar beet	Hsp90	MK095226
HetITS105	*H. medicaginis*	Kearny County, Kansas	Alfalfa	ITS	MK093184
HetITS106	*H. medicaginis*	Kearny County, Kansas	Alfalfa	ITS	MK093185
HetITS107	*H. medicaginis*	Kearny County, Kansas	Alfalfa	ITS	MK093186
HetITS108	*H. medicaginis*	Kearny County, Kansas	Alfalfa	ITS	MK093187
HetITS109	*H. medicaginis*	Kearny County, Kansas	Alfalfa	ITS	MK093188
HetITS110	*H. medicaginis*	Kearny County, Kansas	Alfalfa	ITS	MK093189
HetITS111	*H. medicaginis*	Kearny County, Kansas	Alfalfa	ITS	MK093190
HetITS112	*H. medicaginis*	Kearny County, Kansas	Alfalfa	ITS	MK093191
HetITS113	*H. medicaginis*	Kearny County, Kansas	Alfalfa	ITS	MK093192

### Host testing

Preliminary host testing was conducted at Kansas State University using infested field soil containing an estimated 365 *Heterodera* sp. eggs and infective second-stage juveniles (J2), as well as 325 *Meloidogyne hapla* Chitwood, 1949 J2/100 cm^3^. The soil was placed into 450-cm^3^ D40 Deepots (Stuewe and Sons Inc., Tangent, OR) and planted to either Kansas common alfalfa, an undetermined hybrid of corn, Flyer soybean, or Rutgers tomato. Nematode reproduction was determined after one and two months under greenhouse conditions. *Heterodera* females and cysts were dislodged from roots with water spray and collected on a 250-μm-pore sieve and counted. Vermiform males and J2 of *Heterodera* and *M. hapla* were collected on a 25-μm-pore sieve from one- and two-week incubations of roots in aerated water and counted.

### Morphological and microscopic analysis

Cysts and infective juvenile stages were examined at the USDA MNGDBL and at the University of Nebraska Nematology Laboratory. Select juvenile measurements are presented in Table 2 alongside measurements from Gerber and Maas’s (1982) redescription. Images of juveniles and adult males were taken with a Leica DMLB light microscope with differential interference contrast optics and a Leica DC300 video camera. All juveniles examined at the University of Nebraska were provided an identification number which links specimen images, measurements, and placement on phylogenetic trees. Cysts were prepared for scanning electron microscopy by fixation in a 4% formalin solution followed by a graded series of alcohol to 100% ethyl alcohol prior to critical point drying and coating with gold. Images were obtained on a Hitachi S-3000N scanning electron microscope located in the Morrison Microscopy Core Research Facility at the University of Nebraska.

**Table 2:** Morphological data on juveniles, all measurements in µm.

Specimen No.	Length	Stylet	Tail	Hyaline
1	440	25	–	25
2	421	25	–	21
3	425	25	45	25
4	420	25	50	25
5	421	24	43	22.5
6	420	23	46	23
7	447	25	45	28
8	475	24	52	25.5
9	462	25	45	22.5
10	395	25.5	45	25
11	491	25	–	25.5
12	485	25	50	21
13	413	25	48	25
14	410	26	40	20
15	415	25	45	23
NID 7095	462.5	25	50	27
NID 7096	442.5	24	51	30
NID 7243	477.5	25	52.5	30
NID 7244	455	26	50	25
NID 7245	450	26	49	22.5
NID 7246	425	25	48	27.5
NID 7247	452.5	25	47.5	27.5
NID 8876	472	27	52	28
NID 8877	470	26	50	32
Mean	445.0	25.1	47.8	25.3
SD	27.2	0.8	3.2	3.0
Maximum	491	27	52.5	32
Minimum	395	23	40	20
Gerber and Maas (1982) *n* = 100 *Heterodera medicaginis*	462 (417–512)	25 (24–26)	51.8 (41–60)	28.5 (22–33)
Mulvey and Golden (1983) *H. glycines*	439 ± 6.7 (375–490)	23 ± 0.01 (22–24)	50.4 ± 1.0 (42–59)	26.6 ± 0.7 (20–33)
Mulvey and Golden (1983) *H. schachtii*	452 ± 49.7	25.6 ± 0.11	48.5 ± 0.73	27.1 ± 0.61

### Molecular analyses

The primers used for amplification of the COI gene region were:

- COI-F4a-Het-5′-CAGTTATATAATTCTTTTATTACTAGTCATGCATTAATTATRATTTTTTTTYTRGTTATACC-3′.
- COI-R10b-Het 5′-CCAAAAAAAAAAAAAATCACTATAATCYAAATATTTACGDGG-3′
- The sequencing primers were COI-F4a-Het and for the corresponding strand, an internal primer COI-R8-Het-5′-GAAAATGAGCTACCACATAATAAGTATCATGSARAACMACATCCAAACTAGC-3′.

After removal of the primer sequences, amplification products from the *Heterodera* specimens were 862 base pairs. GenBank sequences used in this study generally were 100 to 300 nucleotides shorter than sequences generated with the new primer set. The ITS1 primer set used in the University of Nebraska Laboratory was reported in the study of Cherry et al. (1997).

### Amplification conditions

Nematodes amplified at the UNL Nematology Laboratory were individually smashed in 18 µL of sterile H_2_O with a transparent microfuge micropipette tip on a coverslip, added to a 0.5 mL microfuge tube and stored at −20°C until needed. Amplification conditions were as follows: denaturation at 94°C for 5 min, followed by 45 cycles of denaturation at 94°C for 30 s, annealing at 48.0°C or 50.0°C for 30 s, and extension at 72°C for 90 s with a 0.5°C per second ramp rate to 72°C. A final extension was performed at 72°C for 5 min as described by Powers et al. (2014) and Olson et al. (2017). PCR products were separated and visualized on 1% agarose using 0.5× TBE and stained with ethidium bromide. PCR products of sufficiently high quality were cleaned and sent for sequencing of both strands by the University of California, Davis DNA Sequencing Facility.

Nematodes analyzed in the Beltsville lab were smashed in worm extraction buffer and extracts prepared as described by Skantar et al. (2012). The ITS and 28S rDNA regions were amplified using primers TW81 and AB28, and D2A and D3B, respectively (Skantar et al., 2012). COI was amplified with primers Het-CoxIF and HetCox-1R according to Subbotin et al. (2017). Partial Hsp90 fragments were amplified with primers U288 and L1110 (Skantar et al., 2012). PCR products were cleaned with the Monarch DNA Gel Exraction Kit (NEB, Ipswich, MA). ITS, COI, and Hsp90 amplicons were cloned using the Strataclone PCR Cloning Kit (Agilent, Santa Clara, CA) according to manufacturer’s instructions. Plasmid clones of DNA were prepared with the Monarch Plasmid Miniprep Kit (NEB) and sequenced by Macrogen, Inc.

### Data storage

Nucleotide sequences have been submitted to GenBank and the Barcode of Life Database (BOLD).

### Phylogenetic analysis

Hsp90 sequences obtained for the Kansas population were aligned with partial Hsp90 genomic DNA sequences from other cyst nematode species (new or from GenBank) using the MAFFT algorithm within Geneious 10.2.6. (https://www.geneious.com). The sequence data set was analyzed with Bayesian interference (BI) using the MrBayes module within Geneious under the model GTR with rate variation set to invgamma, 6 gamma categories, and outgroup set to *Globodera pallida* (Stone, 1973) Behrens, 1975. The Markov-Chain Monte Carlo (MCMC) values were set to 1 × 10^6^ chain length, subsampling frequency 1,000, four heated chains, and a burn-in length of 10,000. Two runs were performed for each analysis. Topologies were used to generate a 50% majority rule consensus tree.

ITS1 and COI phylogenetic trees were constructed under maximum likelihood (ML) criteria in MEGA version 6. Sequences were edited using CodonCode Aligner version 8.0.1 (http://www.codoncode.com/) and aligned using MUSCLE within MEGA version 6 (Tamura et al., 2013). The gap opening penalty was set at −400 with a gap extension penalty of −200. For the COI tree, the General Time Reversible Model with Gamma distributed rates with Invariant sites (GTR + G + I) was determined to be the best substitution model by Bayesian information criterion using the best fit substitution model tool in MEGA 6.0., while the ITS1 tree used HKY. Both ML trees used a ‘use all sites’ option for gaps and 200 bootstrap replications to assess clade support.

## Results

Host trial results indicated that among the crop species tested, only alfalfa was a suitable host for the Kansas alfalfa *Heterodera* population (Table 3). Mature females and cysts were recovered from alfalfa roots at two months after planting, but *Heterodera* J2 were recovered from alfalfa root incubations at both trial periods. In contrast, *Meloidogyne* J2 were recovered in large numbers from tomato roots, with lower numbers recovered from alfalfa and soybean roots.

**Table 3:** Host trial for alfalfa cyst nematodes.

	Crop			
	Alfalfa	Corn	Soybean	Tomato
*Month 1*				
Root weight (g)	0.04	0.29	0.21	0.16
*Heterodera*				
Females and cysts	0	0	0	0
Cysts/g	0	0	0	0
*Heterodera* infective juveniles				
Root incubation	70	0	0	0
J2/g	1,750	0	0	0
*Meloidogyne* J2 infective juveniles				
Root incubation	210	4	10	3,100
J2/g	5,250	14	48	19,375
*Heterodera/Meloidogyne* males				
Root incubation	130	0	20	0
Males/g	3,250	0	95	0
*Month 2*				
Root weight (g)	0.69	0.95	0.54	0.23
*Heterodera*				
Females and cysts	52	0	0	0
Cysts/g	75	0	0	0
*Heterodera* infective juveniles				
Root incubation	292	0	0	0
J2/g	423	0	0	0
*Meloidogyne* J2 infective juveniles				
Root incubation	0	0	308	12,040
J2/g	0	0	570	52,348
*Heterodera/Meloidogyne* males				
Root incubation	136	0	56	520
Males/g	197	0	104	2,261

**Figure 1:**
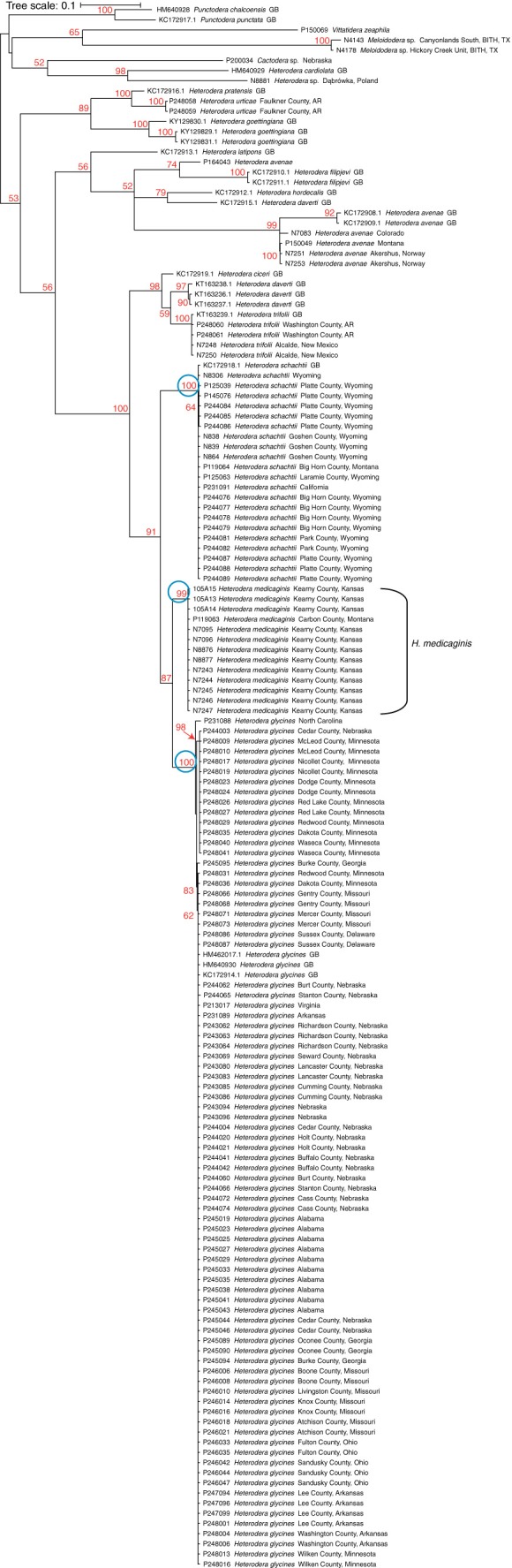
Maximum likelihood tree inferred from the COI gene. Each new specimen is represented by an identification number, species name, and location. GenBank accessions are labeled by accession number, name, and the label ‘GB’ (GenBank). Bootstrap support values (%) are labeled in red, the species nodes of *H. schachtii*, *H. medicaginis*, and *H. glycines* are circled in blue.

Figure 1 presents a maximum likelihood tree based on 862 base pairs of the COI mitochondrial gene from 154 specimens of heteroderid species. In total, 13 sequences from isolates collected from soil underneath alfalfa plantings form a well-supported homogeneous group that is a sister group to *Heterodera glycines*. The specimens from alfalfa are distinct from *H. glycines* at 42 nucleotide sites, with a mean pairwise P-distance (raw distance) of 4.7%. In total, 34 of the 42 nucleotide substitutions are at third-base pair positions in the COI gene. Three substitutions result in amino acid changes. The alfalfa specimens plus *H. glycines* form a group that is paired with *H. schachtii* Schmidt, 1871, with the three species constituting a well-supported clade (91 bootstrap value) that is joined to a second well-supported clade (bootstrap support, 98%) of other members of the *H. schachtii* group that include *H. cicero* Vovlas, Greco, and Di Vito, 1985), *H. daverti* Wouts & Sturhan, 1979, and *H. trifolii* Goffart, 1932. All six species within the *H. schachtii* group form a clade with a bootstrap support value of 100.

**Figure 2:**
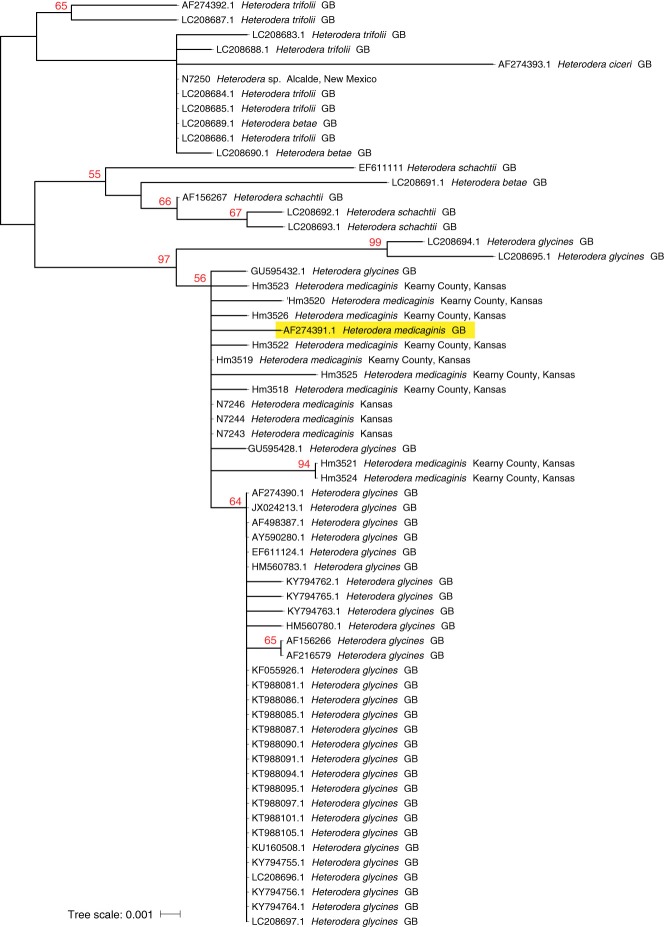
Maximum likelihood tree inferred from the ITS1 gene. Bootstrap support values above 50% are labeled in red. GenBank accession specimen from Russia is boxed in yellow.

The ITS1 tree (Fig. 2) provides less clarity on the distinction between *Heterodera medicaginis* and *H. glycines* due to the sequence heterogeneity within both species. Depending on alignment parameters associated with gap creation and extension for ITS1, the diagnostic signal for this marker may be obscured. Most *H. glycines* sequences retrieved from GenBank cluster together apart, albeit with weak bootstrap support, from the suspected *H. medicaginis* sequences, including the single reference sequence from Russia (AF274391.1). Several GenBank *H. glycines* ITS sequences of questionable identity fall outside the groupings of either species (LC208694.1, LC208695.1).

**Figure 3:**
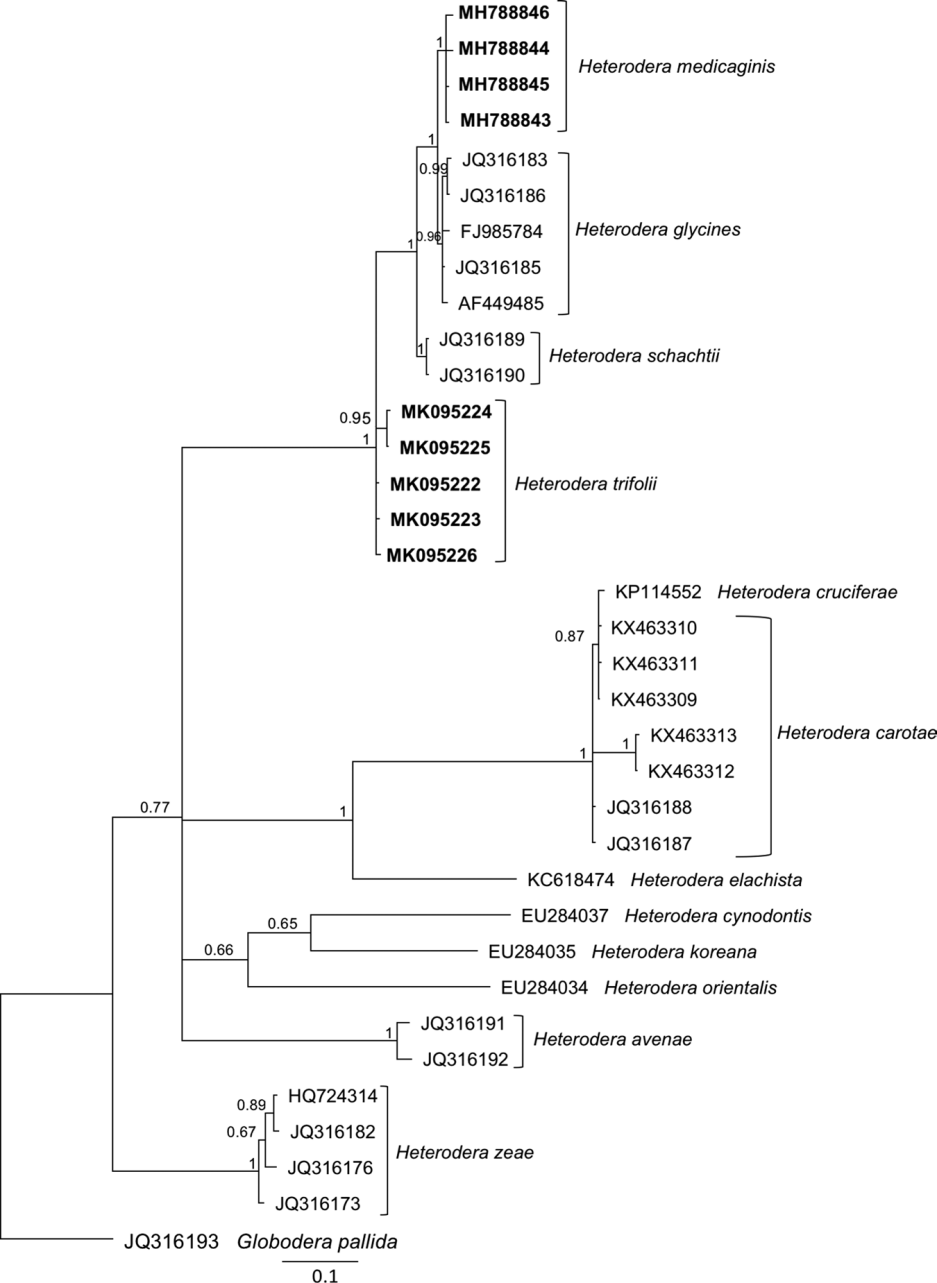
Phylogenetic relationships of *Heterodera* species as inferred from analysis of a partial Hsp90 gene using Bayesian inference. Posterior probabilities more than 50% are given next to nodes. Newly obtained sequences are shown in bold.

Four partial Hsp90 sequences were obtained from the Kansas population (Table 1). Although no Hsp90 sequence was available from a reference population of *H. medicaginis,* Bayesian analysis of partial Hsp90 genomic DNA showed that the Kansas population formed a distinct clade from *H. glycines* and other species from the Schachtii group (Fig. 3), giving further support for identification of this population as *H. medicaginis.*

The 28S sequence from the Kansas population was identical to *H. glycines* GenBank accession numbers (LC208677, KY795945, KY795944, KY795943, KX790324, GU475087, and DQ328692) and therefore did not discriminate *H. medicaginis*. The 28S sequence was submitted to GenBank (Accession No. MH793872) for future phylogenetic studies.

**Figure 4:**
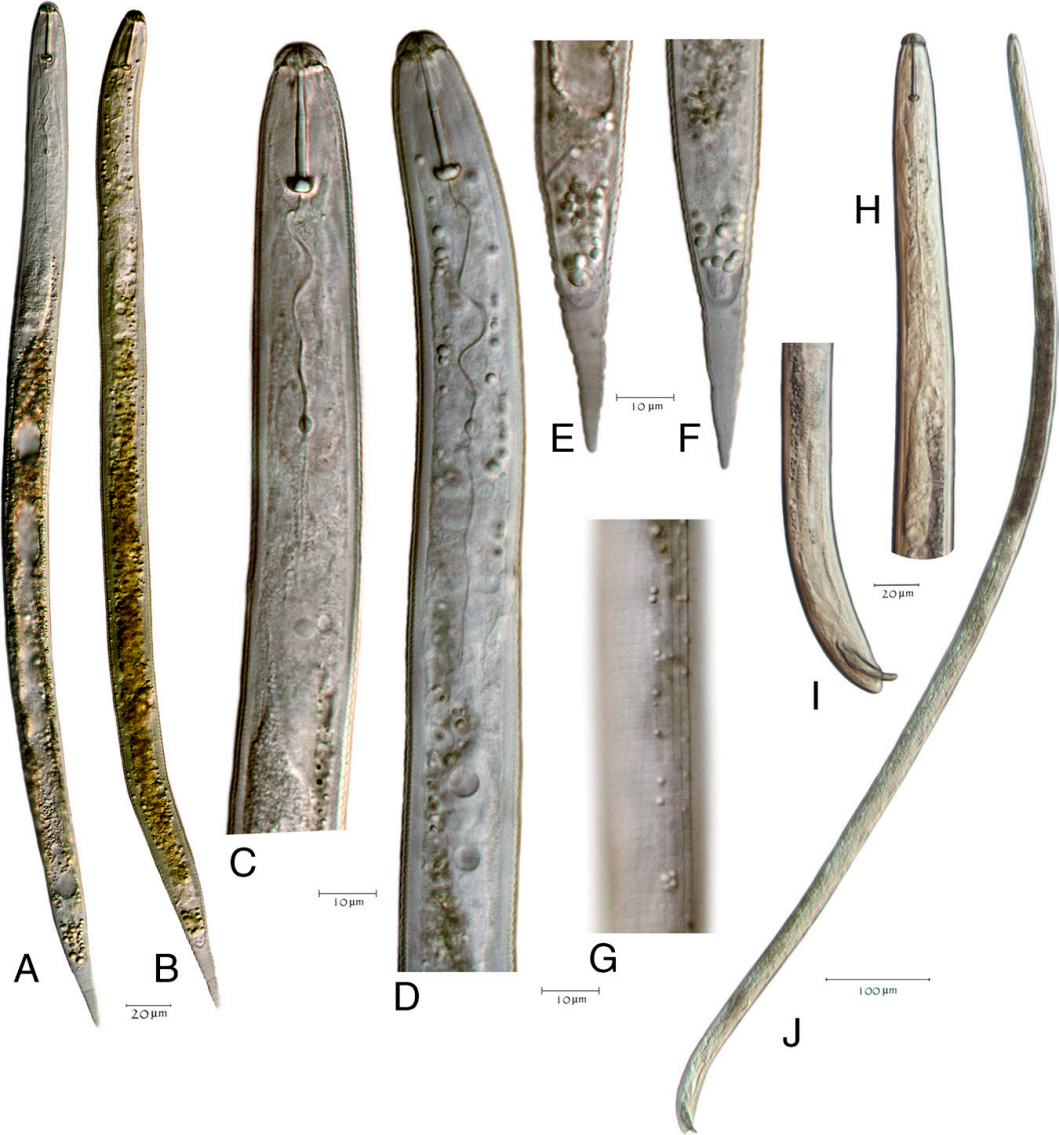
*Heterodera medicaginis* juvenile and male specimens. (A–G), juvenile specimens; (H–J), male specimen. (A) NID 7095, entire body; (B) NID 7243, entire body; (C) NID 7095, anterior; (D) NID 7243, anterior; (E) NID 7095, tail; (F) NID 7243, tail; (G) NID 7243, lateral lines; (H) PNID 169028, anterior; (I) PNID 169028, tail; (J) PNID 169028, entire body.

**Figure 5:**
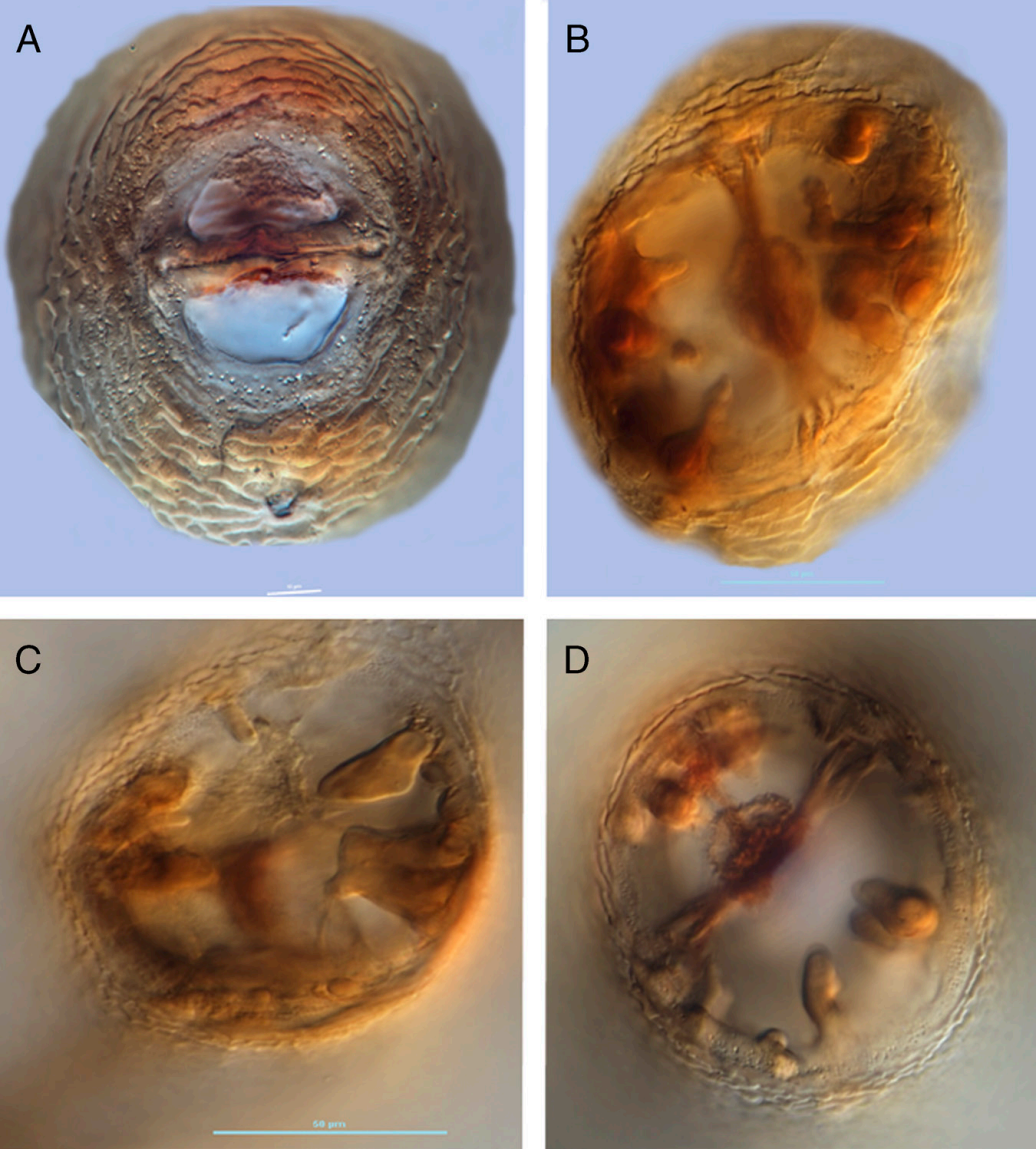
(A–D) light micrograph of vulval cones of *H. medicaginis* showing fenestra (A), bullae (B,D) and underbridge (B,D).

**Figure 6:**
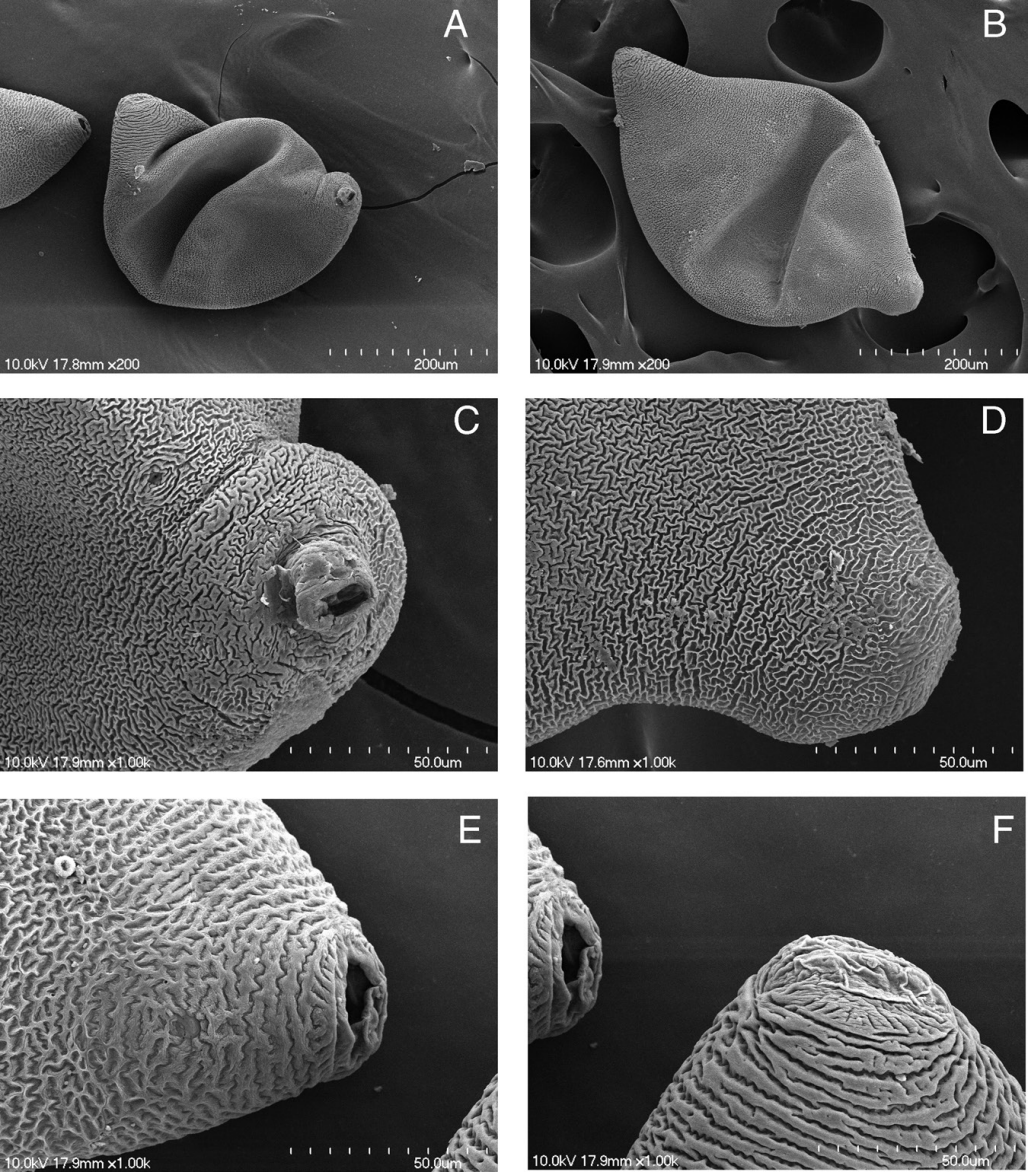
SEM micrographs of *Heterodera medicaginis* cysts from Kansas. (A) entire cyst 1, partial view of cyst 2; (B) entire cyst 3; (C) anterior region and excretory pore of cyst 1; (D) anterior region of cyst 3; (E) vulva and anus of cyst 2; (F) vulva of cyst 2 (left) and cyst 1 (right).

Measurements of J2 specimens are reported in Table 2 and illustrated (Fig. 4A–F). The J2 has four lines in the lateral field and a tail with a finely rounded terminus (Fig. 4E–G). The male anterior end, posterior end, and entire body length showing spicules are also depicted (Fig. 4H–J). Morphologically both cysts and juveniles conformed to *Heterodera medicaginis* except for one cone mount, which displayed molar-shaped bullae typical of *H. schachtii*. That cone, however, was slightly folded. Contrary to the redescription of *H. medicaginis*, the underbridge was moderately well-developed, about 100 mm long, with branches and heavily scattered bullae (Fig. 5B–D). Cysts were mostly oval, wide to lemon-shaped, brown in color, and with a cyst wall displaying a zig-zag pattern with few punctations (Fig. 6C–E). Cysts were ambifenestrate (Fig. 5A), the entire fenestra length ranging from 40 to 47 µm, fenestra width 30 to 32.5 µm or more, vulva slit long with length 38 to 52 µm. The vulva to anus distance was 50 µm (Fig. 6E).

## Discussion

A cyst nematode reproducing on alfalfa has been observed along the Arkansas River in western Kansas. A DNA record from a molecular survey from a Montana alfalfa field suggests the distribution may be wider than a single river valley in Kansas. DNA barcoding data by COI sequence rules out the identity of these cyst nematodes on alfalfa as being *Heterodera glycines*, *H. schachtii*, *H. trifolii*, or either of the two other members of the *H.* ‘schachtii group’. Additionally, the morphology and measurements of juvenile and cyst stages are consistent with those of *H. medicaginis*. Cyst cone structure is more elaborate than was described in Gerber and Maas’s (1982) redescription. Initial greenhouse host-reproduction trials using infested field soil are also consistent with the limited host-range of *H*. *medicaginis*. The presence of *Meloidogyne hapla* in these soils suggests that damage estimates should take both species into consideration. Collectively, these data support the identity of these North American cyst specimens as *Heterodera medicaginis*, the alfalfa cyst nematode. DNA sequence of the COI gene from Russian specimens will be necessary to definitively make the connection between US and confirmed eastern European isolates.
